# Lermoyez Syndrome: A Systematic Review and Narrative Synthesis of Reported Cases

**DOI:** 10.3390/audiolres15040098

**Published:** 2025-08-06

**Authors:** Giorgos Sideris, Leonidas Katsis, Styliani Karle, George Korres

**Affiliations:** 2nd Otolaryngology Department, Attikon University Hospital, National and Kapodistrian University of Athens, 124 62 Athens, Greece; siderisgior@gmail.com (G.S.); leonidas.katsis12@gmail.com (L.K.); stelakarle96@gmail.com (S.K.)

**Keywords:** Lermoyez syndrome, Menière’s disease, endolymphatic hydrops, differential diagnosis of Menière’s disease

## Abstract

**Objectives:** Lermoyez syndrome (LS) is a rare variant of endolymphatic hydrops with a unique clinical presentation characterized by reversible sensorineural hearing loss preceding vertigo. This review aims to synthesize available literature on LS to clarify its clinical characteristics, diagnostic approach, management strategies, and outcomes, and to highlight the distinguishing features from Menière’s disease (MD). **Methods:** A systematic literature review according to PRISMA guidelines was conducted from 1919 to 2025. The extracted data included demographics, symptom profiles, audiovestibular testing, imaging findings, treatment approaches, and patient outcomes. **Results:** A total of 23 studies were identified, reporting 53 individual cases of LS. Patients ranged from 27 to 85 years of age, with a mean age of 50.34 years and a male predominance (64.1%). The hallmark of LS across cases was a reproducible clinical pattern of unilateral low-frequency hearing loss followed by vertigo and subsequent auditory recovery. Audiometry typically confirmed reversible sensorineural hearing loss, while vestibular tests and imaging were often unremarkable, primarily used to exclude alternative diagnoses. Treatment approaches varied and were often based on MD protocols, including dietary modifications, vasodilators, diuretics, and vestibular suppressants. Prognosis was generally favorable, with most patients experiencing both hearing recovery and symptom resolution. **Conclusions:** LS remains a clinically distinct but underrecognized inner ear disorder. Its defining feature—the paradoxical improvement in hearing after vertigo—distinguishes it from Menière’s disease and should prompt clinicians to consider LS in differential diagnosis. Due to the rarity of LS and the lack of standardized guidelines, diagnosis and treatment rely on careful clinical assessment and individualized management strategies.

## 1. Introduction

Lermoyez syndrome (LS) is an uncommon clinical entity within the spectrum of inner ear disorders and is considered a rare variant of Menière’s disease (MD) [1]. Unlike the typical presentation of MD—where episodes of vertigo co-exist with sensorineural hearing loss and tinnitus [2]—LS is characterized by a paradoxical sequence: cochlear symptoms such as hearing loss and tinnitus precede a sudden vertigo attack, after which hearing improves significantly.

This distinctive pattern was first described in 1919 by French otolaryngologist Marcel Lermoyez, who noted that patients experienced “increasing illness, progressive deafness, then sudden vertigo and good hearing” [3]. He hypothesized that the vertigo episode relieved inner ear pressure, thereby restoring auditory function—a theory that has informed subsequent pathophysiological interpretations.

Regarding pathophysiology, LS is widely regarded as a rare endolymphatic hydrops-caused syndrome, marked by abnormal pressure regulation within the membranous labyrinth. The prevailing theory attributes this pattern to an initial rise in endolymphatic pressure affecting cochlear structures, resulting in sensorineural hearing loss and tinnitus. A subsequent release of this pressure—potentially due to micro-rupture of the membranous labyrinth or spontaneous pressure equalization—may trigger vertigo while simultaneously restoring auditory thresholds [4]. This mechanism contrasts with the more progressive auditory decline seen in classical MD.

One proposed mechanism involves transient obstruction within the endolymphatic system, possibly at the ductus reuniens, where displaced saccular debris or otoconia impair fluid communication between cochlear and vestibular compartments [5]. The abrupt resolution of such a blockage during a vestibular episode could normalize endolymphatic flow, explaining both the hearing recovery and onset of vertigo [6].

An alternative hypothesis is the vascular spasm theory, which suggests transient vasospasm of the internal auditory artery may cause reversible cochlear ischemia [7]. Though this model offers a vascular explanation for the auditory fluctuations, it remains less substantiated than pressure-based mechanisms.

Overall, the pathophysiology of LS remains incompletely defined. Current evidence supports a multifactorial model involving mechanical, vascular, and biochemical contributions. Understanding whether LS represents an early stage or a distinct variant of MD remains a topic of ongoing discussion.

This study aims to identify, collect, and analyze all published case reports and case series of LS from both historical and contemporary literature. In addition, it seeks to describe the demographic and clinical profiles of affected individuals, map the diagnostic and therapeutic approaches, and identify patterns in hearing recovery and vertigo recurrence, and evaluate whether LS should be regarded as an early manifestation or a distinct variant of MD.

## 2. Materials and Methods

A systematic review of the literature was conducted to identify and analyze all available case reports and case series describing LS. The search strategy targeted both historical and modern sources, aiming to compile a comprehensive overview of the demographic characteristics, clinical features, diagnostic approaches, treatment modalities, and outcomes associated with this rare condition.

The literature search was performed across five major databases: PubMed, Scopus, Cochrane, and with the help of Google Scholar. Search terms included “Lermoyez syndrome,” “Lermoyez’s disease,” “reverse Menière,” “paradoxical hearing recovery,” and “hydrops with hearing recovery.” Boolean operators and database-specific filters were applied to maximize retrieval sensitivity and specificity. Articles published between 1909 and 2025 were considered for inclusion. No language restrictions were imposed; publications in non-English languages were translated when necessary.

Studies were eligible for inclusion if they presented original clinical case reports or case series explicitly describing LS. To be classified as LS, cases had to feature an initial phase of cochlear symptoms—specifically hearing loss and/or tinnitus—followed by a spontaneous vertigo episode and subsequent recovery of hearing. Studies were excluded if they were literature reviews that did not present new patient data or if the cases described did not adhere to the classical presentation of LS.

From each included study, detailed information was extracted regarding the patient age and sex, clinical features including tinnitus, aural fullness, hearing loss, and vertigo. Audiometric findings before and after vertigo episodes were documented, along with any imaging or vestibular testing performed. Treatment strategies and clinical outcomes—such as hearing recovery, symptom progression, or eventual transition to classical MD—were also recorded. Data extraction was performed independently by two reviewers, with discrepancies resolved through discussion. Due to the heterogeneity of data and small sample sizes, a meta-analysis was not performed. Instead, a narrative synthesis was conducted, grouping cases based on key clinical features (e.g., age, symptom onset, audiometry, treatment outcomes). Missing data were noted and not imputed. The certainty of evidence was not formally graded due to the qualitative nature of data and the reliance on retrospective case reports. As such, findings should be interpreted cautiously.

**Figure 1:**
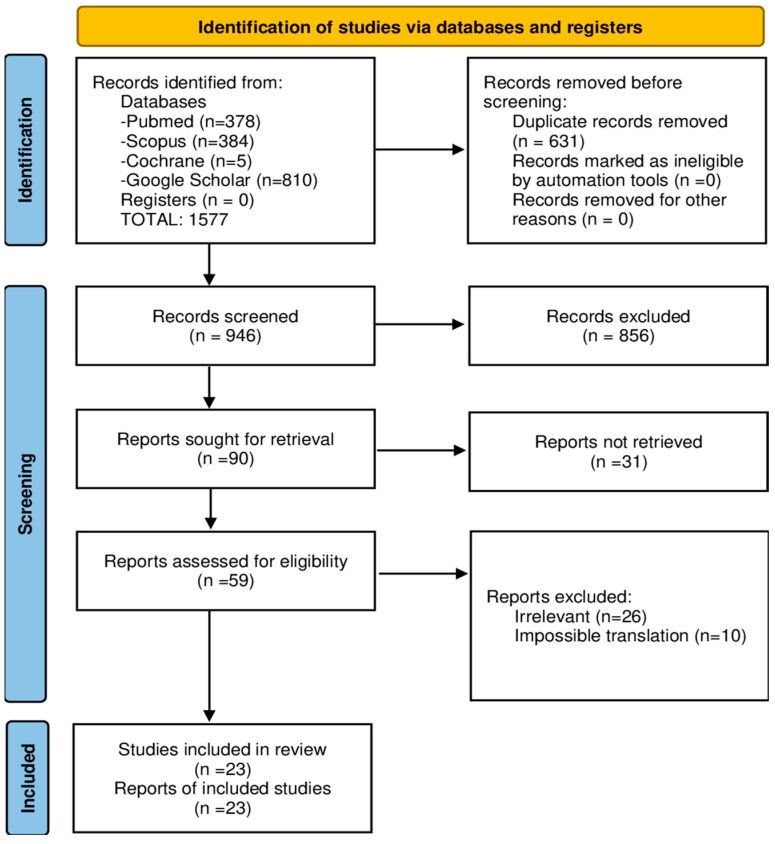
PRISMA flowchart on Lermoyez syndrome studies.

This systematic review was conducted and reported in accordance with the PRISMA (Preferred Reporting Items for Systematic Reviews and Meta-Analyses) 2020 guidelines (Figure 1).

The parameters are described using mean and standard deviation. The predictive accuracy was expressed in the 95% Confidence Intervals (CI). Statistical analysis was performed using IBM SPSS Statistics, version 19. 

## 3. Results

In total, 23 studies were included in this review, reporting 53 individual cases of LS in the literature, spanning over a century—from early historical descriptions to recent clinical reports [8,9,10,11,12,13,14,15,16,17,18,19,20,21,22,23,24,25,26,27,28,29,30]. Among these, 34 patients were males, 17 females, and in 2 cases, sex was not specified. The mean age was 50.34 years (95% CI: 46.35–54.34; range: 27–85 years).

Clinical findings were described in a total of 44 patients. The most consistently reported symptoms were vertigo, present in all cases, followed by sensorineural hearing loss (39 patients), tinnitus (35 patients), and aural fullness (22 cases). Nausea and vomiting accompanied vertigo episodes in 12 patients, while spontaneous or positional nystagmus was noted in 17 patients. In the majority of cases, hearing loss preceded the vertigo attack, which was then followed by a paradoxical improvement in auditory function. Other features such as drop attacks, postural instability, and migraine-like symptoms were noted in a few isolated cases, indicating some degree of heterogeneity in presentation. Audiometric data were available for 46 patients. The predominant finding was low-frequency sensorineural hearing loss, typically involving frequencies from 125 to 1000 Hz, occurring prior to the onset of vertigo. Fluctuating hearing loss over time was observed in seven patients, and progressive high-frequency deterioration was noted in two. Speech discrimination scores were reported in four patients and remained relatively preserved (68–96%) despite considerable shifts in pure-tone thresholds. Glycerol testing was performed in five patients, showing positive responses primarily during early disease stages. Vestibular testing findings were reported in 24 patients. Caloric testing was the most commonly used modality, performed in eight cases, with results ranging from normal responses to unilateral vestibular hypofunction. Cervical Vestibular Evoked Myogenic Potentials (cVEMP) were conducted in four patients, while ocular VEMP (oVEMP) was assessed in three. Video Head Impulse Testing (vHIT) was employed in four patients, typically revealing transient alterations in vestibulo-ocular reflex gain (VOR). Imaging findings were reported in 20 cases. Unilateral endolymphatic hydrops (ELH) were identified in 9 patients who underwent gadolinium-enhanced Magnetic Resonance Imaging (MRI) whereas in the remaining 11 cases, imaging ruled out structural or retrocochlear pathology (Table 1).

Treatment data were available for 25 patients, revealing a primarily conservative management approach. Diuretics such as isosorbide and intravenous furosemide were used in four cases, often in conjunction with glycerol dehydration therapy, especially during acute episodes. Vasodilators like Ronicol and Impletol were administered in two patients, combined with vitamin therapy (Vitamin A or B complex) as part of long-term management. Corticosteroids, either systemic or topical, were included in the treatment regimen for one patient with partial hearing recovery. Additional symptomatic treatments included agents such as diphenidol hydrochloride and cinnarizine to address vertigo. A low-salt diet was documented in one case as part of lifestyle modification.

Outcomes were generally favorable, with most patients experiencing at least partial hearing recovery after vertigo episodes. In 36 patients, hearing thresholds showed significant improvement, with post-attack gains generally ranging from 20 to 30 dB (in 3 patients, hearing improvement occurred during the attack itself, while in the other 33, recovery followed within hours or days). In total, 19 patients recovered completely, while 11 patients had only partial improvement, and 6 patients ultimately developed chronic or progressive hearing loss. Vertigo typically resolved or became less frequent with treatment in the majority of cases, though in a few instances symptoms persisted or evolved into a pattern resembling MD.

## 4. Discussion

### 4.1. Epidemiology

Our analysis identified 53 patients across 23 articles, while an additional 17 patients were cited in 10 publications for which full-text access was not available. While prevalence rates have been estimated at approximately 190 cases per 100,000 individuals, only about 70 individual cases of LS have been documented in the international medical literature over a period spanning more than a century [31]. Shen reported that LS accounts for an estimated 0.2% of cases, or roughly 7 out of more than 4000, underscoring its extreme rarity [8]. This limited recognition likely reflects both its clinical overlap with early-stage MD and the absence of standardized diagnostic criteria. Differentiating LS from MD is essential due to their divergent prognoses, yet it remains challenging in routine practice. Although no definitive demographic pattern has been established, reported cases predominantly involve adults aged 27 to 85 years, with a concentration in the fourth and fifth decades of life and a male predominance (34 out of 53 patients, 64.1%). However, due to the limited number of well-characterized reports and inconsistent data collection, broader epidemiological trends remain uncertain.

### 4.2. Clinical Presentation

#### 4.2.1. Symptoms

Based on clinical data from 41 patients, the hallmark symptom pattern of LS was consistently observed. Sensorineural hearing loss—typically unilateral and affecting low frequencies—preceded vertigo episodes in nearly all cases, with a paradoxical auditory recovery following the vestibular attack [12,17,20,28]. Vertigo was universally reported and often accompanied by nausea, vomiting, and horizontal nystagmus, reflecting peripheral vestibular involvement [8,9,10,23,24,26]. Tinnitus and aural fullness were frequently reported as prodromal symptoms, with tinnitus commonly resolving post-attack [10,13,14,19]. Less commonly, patients experienced migraine-like features, drop attacks, or bilateral involvement [22,25,30], suggesting some heterogeneity in presentation. Nevertheless, the defining clinical feature remains the inversion of the typical Menière’s disease sequence—cochlear symptoms preceding vertigo and followed by hearing recovery. Clinicians should maintain a high index of suspicion when differentiating between these two entities based on clinical presentation (Table 2).

#### 4.2.2. Differential Diagnosis

The differential diagnosis of LS includes: (a) MD that typically involves vertigo that coincides with or follows hearing loss, which tends to fluctuate or worsen over time, without post-vertigo recovery [32], (b) vestibular migraine that may present with vertigo and auditory symptoms but lacks consistent hearing improvement and is often linked to migraine history [33], (c) sudden sensorineural hearing loss (SSNHL) that causes acute hearing loss and imbalance but not the reproducible recovery pattern of LS [34], (d) Perilymphatic fistula that may cause both symptoms, usually in a persistent course [35], and (e) other causes, such as autoimmune inner ear disease or vestibular schwannoma, that should be excluded through imaging and serologic tests [36,37].

### 4.3. Diagnostic Work-Up

#### 4.3.1. Audiological Findings

Audiometry data were reported in most reviewed cases and consistently demonstrated the hallmark profile of LS: low-frequency sensorineural hearing loss preceding vertigo, with thresholds elevated in the 125–1000 Hz range. In several reports, hearing improved significantly within hours to days after the vertigo episode, often returning to near-baseline levels [8,10,14].

Hearing fluctuations were noted across different disease phases. Eggermont et al. and Schoonhoven et al. described cycles of deterioration and recovery primarily affecting low and mid frequencies, while high-frequency thresholds either remained stable or showed progressive decline [18,19]. In some cases, pure-tone audiometry revealed flat or high-frequency loss prior to attacks, and glycerol testing showed variable benefit—more pronounced in early stages and reduced over time [11,29].

Speech discrimination scores were generally preserved despite threshold shifts, indicating transient cochlear dysfunction [12,15]. Repeat audiometry after vertigo episodes frequently confirmed partial or full threshold normalization, reinforcing the hypothesis of reversible endolymphatic pressure dynamics as a likely underlying mechanism.

#### 4.3.2. Vestibular Tests

Vestibular test findings in LS, though less consistently reported than audiometric data, offer valuable insight into the disorder’s fluctuating pathophysiology. Vestibular tests showed transient unilateral hypofunction in several patients, supporting pressure-related dysfunction, as detailed above. Shen et al. reported reduced responses in 5 of 8 affected ears, suggesting transient unilateral hypofunction [8], while Maier et al. and Zhou et al. described canal paresis or asymmetrical vestibular responses [9,10]. In contrast, Zhang et al. and Stoecklin documented normal caloric findings, reflecting episodic rather than permanent dysfunction [11,26].

VEMPs further demonstrated variability. Shen et al., Zhou et al., and Kordiš et al. noted reduced or absent cVEMP and oVEMP responses during symptomatic phases [8,10,27]. vHIT, reported in a few cases, generally showed preserved VOR gains, with transient reductions during acute attacks. Manzari et al. documented spontaneous nystagmus and intact postural control, reinforcing the reversible nature of vestibular involvement [14].

Additional tests, such as electronystagmography (ENG) and electrocochleography (ECochG), used by Schmidt et al. and Masutoshi et al., indicated cochleo-vestibular hydrops in selected cases [15,30]. Overall, the findings support a pattern of transient, often unilateral vestibular dysfunction, aligning with dynamic endolymphatic pressure changes as a key mechanism in LS.

#### 4.3.3. Imaging Findings

MRI, particularly with high-resolution fluid-attenuated inversion recovery (FLAIR) sequences, is primarily employed in LS to exclude retrocochlear or structural inner ear pathologies. In most cases, standard CT and MRI scans are unremarkable, functioning chiefly as exclusion tools rather than providing direct diagnostic evidence. This pattern was observed in reports by Maier et al., Manzari et al., and Kordiš et al. [9,14,27], as well as in earlier studies using petrous bone radiographs, such as those by Schmidt and Stoecklin [12,15,26]. A notable exception is the study by Zhou et al., in which gadolinium-enhanced MRI revealed unilateral ELH in all patients, with moderate cochlear ELH in 66.7% and significant vestibular ELH in 77.8% [10]. These findings lend support to the hypothesis that LS shares underlying fluid pressure dysregulation mechanisms with MD. Although advanced imaging may reveal ELH in rare cases, its diagnostic yield remains low, reinforcing the need for clinical pattern recognition. Despite these limitations, Table 3 summarizes MRI findings that may assist in differentiating LS from MD in select clinical contexts.

### 4.4. Treatment and Outcomes

#### 4.4.1. Management

Several authors have documented a variety of therapeutic strategies for managing LS, reflecting individualized, symptom-based approaches. Zhang et al. [11] administered vasodilators and microcirculation enhancers during the first year of symptoms, while Takeda et al. [17] and Koizuka et al. [20] employed dehydration therapy with glycerol and intravenous furosemide to reduce endolymphatic pressure. Eggermont et al. [18] used cinnarizine for vestibular symptom control, and Schoonhoven et al. [19] advocated dietary sodium restriction, echoing MD protocols. Stoecklin [26] and Takeda et al. [28] combined vitamin A (Arovit) with vasodilators (Ronicol, Impletol), aiming to enhance cochlear microcirculation. Satoshi et al. [29] described a multimodal approach with isosorbide, corticosteroids, and vitamins, while Masutoshi et al. [30] used diphenidol hydrochloride and isosorbide for acute symptom relief.

While many treatments described in historical reports reflect outdated medical practices, they underscore the absence of standardized protocols. As discussed, treatment is largely empirical and based on MD strategies (low-sodium diet, stress reduction, avoidance of caffeine and alcohol, diuretics, betahistine, and corticosteroids—administered systemically or transtympanically—especially when hearing recovery is incomplete and vestibular suppressants and antiemetics are typically used during acute episodes). Surgical interventions (endolymphatic sac decompression, labyrinthectomy) have not been applied in reported LS cases to date.

#### 4.4.2. Prognosis

Outcomes across the reported cases reveal a heterogeneous yet generally favorable clinical trajectory. In several studies, patients demonstrated full or substantial improvement in hearing—especially at low and mid frequencies—following vertigo episodes, consistent with the hypothesis of transient pressure-related cochlear dysfunction in LS [8,9,10]. Zhang et al. [11] observed stable auditory thresholds over long-term follow-up, while other authors noted persistent high-frequency hearing loss in some cases, suggesting incomplete recovery [18,30]. Vestibular symptoms also tended to resolve, with chronic imbalance or progression to MD reported only rarely, as in the cases by Young et al. and Xenellis et al. [16,21]. Although the prognosis of LS is considered favorable—when compared to classic MD—and most patients experience symptom resolution and hearing restoration, a minority exhibit residual or progressive deficits, underscoring the need for long-term follow-up.

#### 4.4.3. Limitations

This systematic review has several limitations that should be acknowledged. Most included studies were retrospective case reports or small case series, lacking standardized diagnostic criteria and consistent outcome measures. Due to this heterogeneity, a meta-analysis was not feasible, and a narrative synthesis was used instead. Risk of bias and reporting bias could not be formally assessed, and some cases lacked key clinical data. These factors may affect the reproducibility and generalizability of the findings.

## 5. Conclusions

Lermoyez syndrome remains a rare and underrecognized variant of endolymphatic hydrops, with fewer than 60 well-documented cases reported over more than a century. Characterized by a paradoxical sequence of auditory and vestibular symptoms—where hearing loss precedes vertigo and is often followed by auditory recovery—LS stands in contrast to the typical presentation of Menière’s disease. Although the pathophysiological mechanisms remain incompletely understood, theories involving transient endolymphatic obstruction and pressure regulation are the most widely accepted. Current diagnostic approaches rely heavily on clinical suspicion, audiovestibular testing, and exclusion of alternative causes through imaging. Treatment strategies are mostly extrapolated from Menière’s disease management and vary widely across reported cases, with no standardized protocol currently in place. Despite the heterogeneity in clinical presentation and treatment, most patients demonstrate a favorable prognosis, particularly in terms of auditory recovery. Improved recognition and reporting of LS cases may not only refine diagnostic criteria but also inform more tailored and evidence-based therapeutic approaches.

## Figures and Tables

**Table 1:** Summary of findings from 23 studies on Lermoyez syndrome.

		Total (%) *n* = 53 Patients
Sex	Males	34
	Females	17
	N/A	2
Age (years)	Mean ± SD	50.34 ± 12.66
	95% CI	46.35–54.34
Clinical symptoms (44/53 patients)	Vertigo	44
	Sensorineural hearing loss	39
	Tinnitus	35
	Aural fullness	22
	Nausea and vomiting	12
	Nystagmus	17
Audiometry (46/53 patients)	Hearing improved during the attack	3
	Hearing recovered after the attack	33
	Fluctuating hearing loss over time	7
	Progressive high-frequency hearing loss	2
	Preserved speech discrimination scores	4
Vestibular testing (24/53 patients)	Glycerol testing performed	5
	Caloric testing	5
	cVEMP testing	4
	oVEMP testing	3
	vHIT testing	4
	Nystagmus documentation	7
Imaging findings (20/53 patients)	Hydrops	9
	Normal	11

**Table 2:** Clinical differences between Menière’s disease and Lermoyez syndrome.

	Menière’s Disease	Lermoyez Syndrome
Sequence of symptoms	Vertigo → Hearing loss/Tinnitus	Hearing loss/Tinnitus → Vertigo
Hearing after the episode	Often worsens or remains the same	Temporarily or fully improves
Permanent hearing loss	Gradual, with repeated episodes	Less likely, especially in early stages
Aural fullness	Common	Common
Tinnitus	Often present and persistent	Present before vertigo, often reduced after
Pathophysiology	Endolymphatic hydrops	Endolymphatic hydrops with possible sudden pressure release
Diagnostic challenge	Recognizable through typical episode pattern	Harder to diagnose due to reversed symptom sequence
Treatment	Diuretics, betahistine, dietary changes, intratympanic injections	Similar approach, focused on preventing recurrent episodes
Prevalence	More common (especially in middle-aged adults)	Very rare

**Table 3:** MRI findings in Menière’s disease vs. Lermoyez syndrome.

	Menière’s Disease	Lermoyez Syndrome
Endolymphatic hydrops	Persistent; usually visible on MRI	Mild or transient; may be absent if MRI is delayed
Cochlear hydrops	Moderate to severe	Mild to moderate (~78% of cases)
Vestibular hydrops	Frequently present (saccule, utricle)	Less frequent (~22% of cases)
Correlation with symptoms	Consistent with chronic vertigo and progressive hearing loss	Consistent with reversible hearing loss
Timing	Less critical—hydrops tends to persist	Very important—ideally performed shortly after symptoms
Technique	3D-FLAIR or HYDROPS protocol with IV gadolinium (4 h delay)	Same technique—requires optimal timing
Diagnostic value	Reliable for assessing severity and disease progression	Requires careful clinical correlation and timing

## Data Availability

No new data were created in this systematic review. Data sharing is not applicable to this article.

## Abbreviations

The following abbreviations are used in this manuscript:

LS	Lermoyez syndrome
cVEMP	Cervical Vestibular Evoked Myogenic Potentials
oVEMP	Ocular Vestibular Evoked Myogenic Potentials
MD	Menière’s disease
vHIT	Video Head Impulse Testing
VOR	Vestibulo-ocular reflex gain
MRI	Magnetic Resonance Imaging
SSNHL	Sudden sensorineural hearing loss
ENG	Electronystagmography
ECochG	Electrocochleography
ELH	Endolymphatic hydrops
FLAIR	Fluid-attenuated inversion recovery
